# Secuenciación del SARS-CoV-2: la iniciativa tecnológica para fortalecer los sistemas de alerta temprana ante emergencias de salud pública en Latinoamérica y el Caribe

**DOI:** 10.7705/biomedica.5841

**Published:** 2020-11-15

**Authors:** Diego A. Álvarez-Díaz, Katherine Laiton-Donato, Carlos Franco-Muñoz, Marcela Mercado-Reyes

**Affiliations:** 1 Unidad de Secuenciación y Genómica, Dirección de Investigación en Salud Pública, Instituto Nacional de Salud, Bogotá, Colombia Instituto Nacional de Salud Bogotá Colombia; 2 Grupo de Salud Materna y Perinatal, Dirección de Investigación en Salud Pública, Instituto Nacional de Salud, Bogotá, Colombia Instituto Nacional de Salud Bogotá Colombia; 3 Grupo de Parasitología, Dirección de Investigación en Salud Pública, Instituto Nacional de Salud, Bogotá, Colombia Instituto Nacional de Salud Bogotá Colombia; 4 Dirección de Investigación en Salud Pública, Instituto Nacional de Salud, Bogotá, Colombia Instituto Nacional de Salud Bogotá Colombia

**Keywords:** infecciones por coronavirus, síndrome respiratorio agudo grave, virus del SRAS, secuenciación de nucleótidos de alto rendimiento, vigilancia epidemiológica, políticas en salud pública, Coronavirus infections, viral isolation severe acute respiratory syndrome, SARS virus, high-throughput nucleotide sequencing, epidemiological surveillance, public health policies

## Abstract

La pandemia de COVID-19 causada por el SARS-CoV-2 es un problema de salud pública sin precedentes en los últimos 100 años, así como la respuesta centrada en la caracterización genómica del SARS-CoV-2 prácticamente en todas las regiones del planeta. Esta pandemia surgió durante la era de la epidemiología genómica impulsada por los continuos avances en la secuenciación de próxima generación. Desde su reciente aparición, la epidemiología genómica permitió la identificación precisa de nuevos linajes o especies de agentes patógenos y la reconstrucción de su variabilidad genética en tiempo real, lo que se hizo evidente en los brotes de influenza H_1_N_1_, MERS y SARS. Sin embargo, la escala global y descontrolada de esta pandemia ha generado una situación que obligó a utilizar de forma masiva herramientas de la epidemiología genómica como la rápida identificación del SARS-CoV-2 y el registro de nuevos linajes y su vigilancia activa en todo el mundo. Antes de la pandemia de COVID-19 la disponibilidad de datos genómicos de agentes patógenos circulantes en varios países de Latinoamérica y el Caribe era escasa o nula. Con la llegada del SARS-CoV-2 dicha situación cambió significativamente, aunque la cantidad de información disponible sigue siendo escasa y, en países como Colombia, Brasil, Argentina y Chile, la información genómica del SARS-CoV-2 provino principalmente de grupos de investigación en epidemiología genómica más que como producto de una política o programa de vigilancia en salud pública. Ello evidencia la necesidad de establecer políticas de salud pública orientadas a la implementación de la epidemiología genómica como herramienta para fortalecer los sistemas de vigilancia y alerta temprana frente a amenazas para la salud pública en la región.

## Abstract (en)

The COVID-19 pandemic caused by SARS-CoV-2 is a public health problem on a scale unprecedented in the last 100 years, as has been the response focused on the rapid genomic characterization of SARS-CoV-2 in virtually all regions of the planet. This pandemic emerged during the era of genomic epidemiology, a science fueled by continued advances in next-generation sequencing. Since its recent appearance, genomic epidemiology included the precise identification of new lineages or species of pathogens and the reconstruction of their genetic variability in real time, evidenced in past outbreaks of influenza H1N1, MERS, and SARS.

However, the global and uncontrolled scale of this pandemic created a scenario where genomic epidemiology was put into practice *en masse,* from the rapid identification of SARS-CoV-2 to the registration of new lineages and their active surveillance throughout the world. Prior to the COVID-19 pandemic, the availability of genomic data on circulating pathogens in several Latin America and the Caribbean countries was scarce or nil. With the arrival of SARS-CoV-2, this scenario changed significantly, although the amount of available information remains scarce and, in countries such as Colombia, Brazil, Argentina, and Chile, the genomic information of SARS-CoV-2 was obtained mainly by research groups in genomic epidemiology rather than the product of a public health surveillance policy or program. This indicates the need to establish public health policies aimed at implementing genomic epidemiology as a tool to strengthen surveillance and early warning systems against threats to public health in the region.

El mundo enfrenta la devastadora pandemia de COVID-19 causada por el betacoronavirus SARS-CoV-2, reportado por primera vez en la ciudad de Wuhan en China en diciembre de 2019. Al final de septiembre de 2020 el virus se había dispersado a más de 180 países, con más de 33 millones de casos y un millón de muertes en todo el mundo ^1^. Mientras el SARS-CoV-2 se dispersaba por el Viejo Mundo, los países latinoamericanos se preparaban para disminuir la exposición y el impacto en la población.

El entrenamiento de profesionales de la salud y la adquisición de insumos y equipos para el diagnóstico y la caracterización genética de SARS-CoV-2 resultó esencial para la toma de decisiones enfocadas en la contención y la posterior mitigación de la enfermedad ^2^. No obstante, casi un mes después de detectarse el primer caso latinoamericano de COVID-19 en Brasil, se confirmaron por miles los casos de la enfermedad en la mayoría de los países de Latinoamérica y el Caribe ^3^. Para septiembre de 2020, países como Brasil, México, Perú, Chile y Colombia habían superado las cifras de casos reportados de la mayoría de países del Viejo Mundo ^1^, convirtiéndose en el nuevo foco de la pandemia, por lo que se espera un incremento de los casos y, consecuentemente, un mayor impacto de la COVID-19 en Latinoamérica.

Además del impacto en la salud, las medidas de aislamiento preventivo llevaron a una recesión económica que ha exacerbado la desigualdad social en regiones en desarrollo, incluida Latinoamérica, donde se estima que la cifra de personas pobres y en pobreza extrema aumentará de 185 a 230 millones y de 68 a 96 millones, respectivamente, en lo que constituye la peor recesión económica en un siglo en la región ^4^.

En este sentido, la pandemia de COVID-19 representa un obstáculo significativo para el cumplimiento de algunos de los Objetivos de Desarrollo Sostenible como el de poner fin a la pobreza en todas sus formas en todo el mundo, garantizar una vida sana y promover el bienestar para todos en todas las edades. Además, dejó en evidencia la necesidad de aumentar la inversión en investigación científica, así como de mejorar la capacidad tecnológica en los sistemas de alerta temprana y gestión de riesgos frente a epidemias en Latinoamérica.

En este contexto, los gobiernos se enfrentan a uno de los mayores retos ante la necesidad de minimizar las muertes por COVID-19 y, al mismo tiempo, reducir el impacto económico de la propagación del virus en los territorios ^5^. Entre las estrategias para el manejo de la pandemia se han implementado medidas como el aumento del número de pruebas de diagnóstico molecular y de unidades de cuidados intensivos.

Por último, la acumulación de información genómica sobre el SARS-CoV-2 circulante en diferentes países de Latinoamérica y el Caribe, en su mayoría producto de esfuerzos locales de implementación de tecnologías de secuenciación de próxima generación, representa una herramienta de gran utilidad para fortalecer la vigilancia epidemiológica y la adopción acertada de decisiones en salud pública para controlar este virus y los demás agentes infecciosos que circulan en la región. Sin embargo, la aplicación de este enfoque en nuestra región implica enfrentar retos importantes que van desde el establecimiento de políticas regionales en salud pública para fortalecer la cooperación entre los laboratorios de salud pública y facilitar la transferencia de tecnología y entrenamiento, además de una inversión de recursos económicos importantes para la adquisición de insumos, infraestructura y mejoramiento de la capacidad instalada en el laboratorio.

## Secuenciación del SARS-CoV-2 y su papel en la adopción de decisiones en salud pública

Las tecnologías de secuenciación de próxima generación implementadas de rutina para la vigilancia de agentes patógenos en agencias de salud pública en Europa *(Public Health England)* y Norteamérica *(Centers for Disease Control and Prevention, Public Health Agency of Canada),* han permitido la identificación específica de focos de transmisión, que aportan información crucial para la adopción de decisiones basadas en la evidencia para prevenir la dispersión de enfermedades como la tuberculosis ^6^. Además, la vigilancia genómica permitió la identificación del SARS-CoV-2 como una nueva especie viral pocas semanas después del primer brote en China -algo que tradicionalmente tomaba varios meses-, lo que encendió las alarmas que llevaron al inicio de cuarentenas en todo el mundo ^7^.

La implementación de las tecnologías de secuenciación de próxima generación en la vigilancia de agentes patógenos posibilita el desarrollo de análisis basados en la epidemiología genómica que, en el marco de la presente pandemia de COVID-19, busca caracterizar el componente genético de las cepas de SARS-CoV-2 circulantes y su correlación con datos epidemiológicos registrados a partir del seguimiento de casos y contactos. En este sentido, la iniciativa global de compartir todos los datos de influenza (GISAID) ^1^, brinda la oportunidad de mejorar significativamente los sistemas de alerta temprana de cualquier país interesado en implementar la epidemiología genómica como parte de sus políticas de salud pública. Además, es importante resaltar el aporte técnico de iniciativas disponibles en la web que permiten acceder a protocolos estandarizados para la secuenciación masiva del SARS-CoV-2 ^8^.

**Figura 1:**
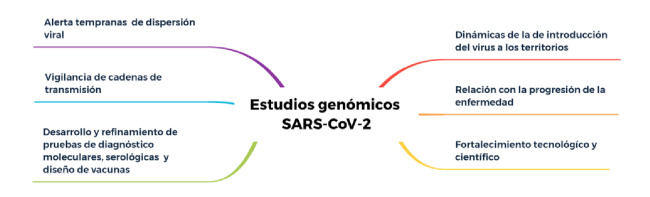
Aplicaciones de los estudios genómicos de SARS-CoV-2

Con este enfoque, a medida que el virus se diseminaba por el mundo fue posible evidenciar rápidamente la acumulación de cambios en su genoma, lo que tuvo un impacto directo en el desarrollo y el refinamiento de métodos de diagnóstico molecular y serológico, además de convertirse en el principal insumo para el diseño racional de candidatos a vacunas y tratamientos. Los primeros datos de vigilancia genómica provenientes de países suramericanos como Argentina, Brasil, Colombia, Chile, Ecuador y Uruguay, han aportado evidencia útil para la selección de métodos de diagnóstico molecular y serológico, así como para la formulación de vacunas ^9^^,^^10^, en la que la variabilidad genética de las cepas del SARS-CoV-2 circulantes en la región ha sido determinante (figura 1).

## Lecciones aprendidas en epidemiología genómica de otros virus endémicos en Latinoamérica

En Latinoamérica, la circulación simultánea de varios virus transmitidos por mosquitos (Arbovirus), entre ellos los relacionados con las recientes epidemias de dengue, Zika y chikungunya, representa una amenaza para la salud pública con implicaciones sociales y económicas de enorme importancia ^11^^,^^12^, lo que se suma a la presencia de otros virus emergentes, como el Mayaro y el Oropuche, cuyo impacto todavía no se ha estimado. Algunos países latinoamericanos han aportado muy buenos ejemplos sobre la utilidad de los datos genómicos de los arbovirus en la comprensión de fenómenos epidemiológicos de importancia en salud pública; entre ellos cabe destacar la posibilidad de determinar el retraso entre el momento de la aparición de un arbovirus y la primera detección en un área geográfica, la distribución espacial de las cepas de mayor virulencia, la transmisibilidad de vectores y el mayor riesgo de fracaso de eventuales vacunas ^13^. En Colombia, la vigilancia genómica de arbovirus permitió identificar los genotipos del dengue, chikungunya y Zika, así como la diversificación regional de linajes de estos virus en el país ^14^^-^^16^.

**Figura 2:**
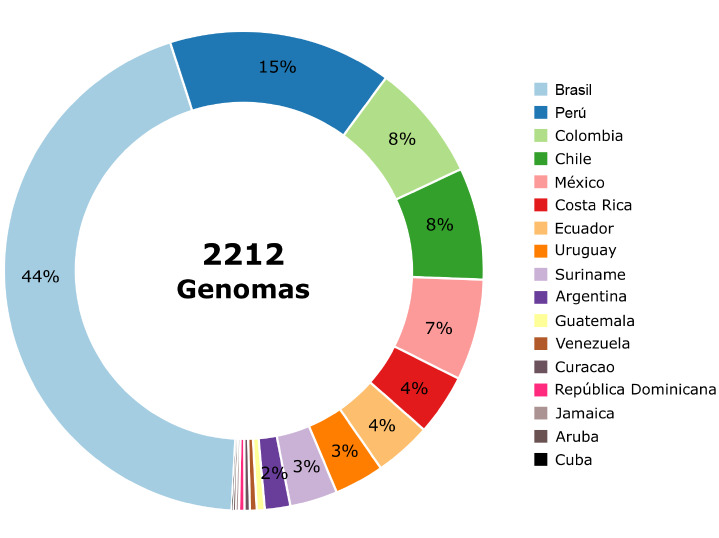
Distribución de los genomas del SARS-CoV-2 reportados en la base de datos GISAID originados en países de Latinoamérica y el Caribe

Brasil ha sido pionero en la generación de datos, así como en el acceso a la infraestructura y el personal capacitado en la producción de datos genómicos, algo que quedó evidenciado por el hecho de haber reportado el 40 % de los genomas de SARS-CoV-2 en Latinoamérica. Sin embargo, con la pandemia de COVID-19 en la región, varios países de Latinoamérica demostraron capacidad técnica para contribuir a la vigilancia genómica del SARS-CoV-2, pese a que la cantidad de genomas reportados fue limitada (figura 2).

## Secuenciación del SARS-CoV-2 en Latinoamérica: hacia la implementación de una red de vigilancia genómica

El primer genoma de SARS-CoV-2 se reportó el 13 de enero de 2020 y hacia finales de septiembre del 2020 se habían reportado en la base de datos GISAID más de 126.000 genomas a nivel mundial, de los cuales más de 2.200 provienen de Latinoamérica y el Caribe. La abundancia y la velocidad de la producción de datos genómicos en el contexto de la epidemia es un hecho sin precedentes en la historia de la humanidad y ha permitido el monitoreo en tiempo real del avance de la pandemia, el estudio de las introducción del virus en diferentes territorios ^17^, la vigilancia de la utilidad y aplicabilidad de los métodos diagnósticos y el desarrollo de las posibles vacunas ^9^^,^^18^.

**Figura 3:**
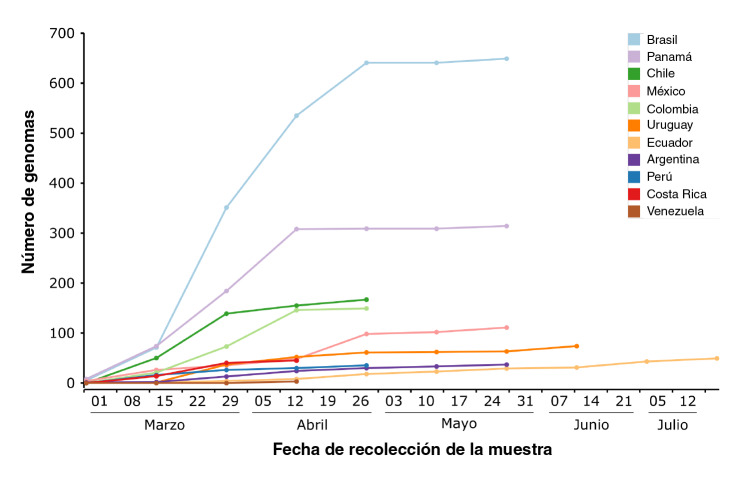
Distribución temporal de los reportes de genomas de SARS-CoV-2 en países de Latinoamériva y el Caribe utilizando la fecha de recolección de la muestra asociada con la secuencia reportada en GISAID

Además, la información temprana aportada por países como Brasil, Panamá, Chile y Colombia, sobre la composición genética de las cepas de SARS-CoV-2 que llegaron durante los primeros meses (figura 3) fue crucial para monitorear la diversificación regional, la fijación de linajes y la importación entre países vecinos a la región ^17^^,^^19^.

Por otro lado, el estudio genómico del SARS-CoV-2 permitió identificar los cambios acumulados en el genoma del virus desde su origen en China, su dispersión por el mundo y la llegada a Latinoamérica. Entre los cambios más frecuentes, la mutación D614G en la proteína S del SARS-CoV-2 ha sido de particular interés, dado que su presencia está asociada con el linaje B.1, dominante en la pandemia, el cual virtualmente reemplazó al linaje A.1, su predecesor. Dicha observación orientó los estudios sobre la asociación de esta mutación con una mayor dispersión e infectividad del virus ^20^^,^^21^ y una antigenicidad reducida, lo que ha llevado a considerar la posibilidad de adaptar las vacunas y las pruebas serológicas ^22^.

En Suramérica, la secuenciación del SARS-CoV-2 permitió establecer que más del 80 % de los genomas reportados para la región presentaban la sustitución D614G ^10^, información valiosa sobre la historia natural del virus que puede orientar a los responsables de las decisiones en salud pública para la selección, implementación o refinamiento de métodos de diagnóstico, vacunas y tratamientos (cuadro 1). Esto evidencia la necesidad de generar políticas públicas para aumentar los recursos estatales dedicados a implementar la secuenciación genómica como una herramienta de vigilancia temprana de rutina ante eventos de interés en salud pública en Latinoamérica y el Caribe.

**Cuadro 1:** Estudios de epidemiología genómica realizados en Latinoamérica y el Caribe durante la pandemia ocasionada por el SARS-CoV-2

Estudio	Aproximación	País
Jesus, *et al*., 2020 ^23^	Descripción genómica de SARS-CoV-2 del primer caso de COVID-19 reportado en Brasil y casos asociados. Los genomas obtenidos se relacionaban con secuencias reportadas en Italia.	Brasil
Candido, *et al*., 2020 ^24^	Estudio que combina la epidemiología y la genómica para investigar el impacto de intervenciones no farmacológicas en el control de la pandemia en Brasil. Los datos genómicos permitieron detectar más de 100 eventos de introducción de SARS-CoV-2.	Brasil
Resende, *et al*., 2020 ^19^	Estudio de vigilancia genómica de la diversidad de SARS-CoV-2 durante la fase temprana de la epidemia en Brasil. Se encontraron múltiples introducciones del virus y evidencia de transmisión comunitaria, y se determinó el linaje B.1.1, lo que sugiere que fue exportado a otros países de Latinoamérica.	Brasil
Xavier, *et al*., 2020 ^25^	Estudio genómico de SARS-CoV-2 en el estado de Minas Gerais. Se analizó la información de 40 genomas y se encontró evidencia de múltiples introducciones del virus y se determinó una posible fecha de introducción en febrero desde Italia, congruente con la evidencia epidemiológica.	Colombia
Álvarez-Díaz, *et al*., 2020 ^9^	Descripción de la variabilidad genética de los genomas colombianos del SARS-CoV-2 en las regiones de hibridación de los oligonucleótidos de los principales métodos descritos a nivel mundial para la detección molecular	Colombia
Franco-Muñoz, *et al*., 2020 ^10^	Descripción de la frecuencia de sustitución de las proteínas S y N del SARS-CoV-2 en Suramérica. Las sustituciones D614G en S y R203K / G204R en N fueron las más frecuentes en Suramérica, observadas en el 83 y 34 % de las secuencias, respectivamente.	Colombia
Laiton-Donato, *et a*l., 2020 ^17^	Estudio de la emergencia y las rutas de importación de COVID-19 a Colombia utilizando observaciones epidemiológicas, históricas de viajes aéreos y análisis filogenéticos. Se proporcionó evidencia de múltiples introducciones, principalmente de Europa con, por lo menos, 12 linajes circulantes.	Colombia
López-Álvarez, *et al*., 2020 ^26^	Reporte de la secuencia del genoma de un aislamiento viral de un paciente con COVID-19 que estaba infectado en Cali, Colombia. El paciente no tenía antecedentes de viajes recientes y no requirió hospitalización	Colombia
Paniz-Mondolfi, *et al*., 2020 ^27^	Descripción de primeros genomas del SARS-CoV-2 en la región fronteriza colombo-venezolana. Los genomas del SARS-CoV-2 de Venezuela fueron clasificados en el linaje B1, linaje B.1.13.	Colombia
Castillo, *et a*l., 2020 ^28^	Estudios de genomas completo de los primeros cuatro casos de la nueva enfermedad por coronavirus en Chile en pacientes que viajaron a Europa y el sureste asiático	Chile
Márquez, *et al*., 2020 ^29^	Estudio metagenómico de tres casos que confirmaron la presencia de SARS-CoV-2 en coexistencia con bacterias patógenas que sugieren coinfección con *Streptococcus pneumoniae*, *Mycobacterium tuberculosis*, *Staphylococcus aureus* y *Chlamydia* spp.	Ecuador
Garcés-Ayala, *et al*., 2020 ^30^	Descripción de la secuencia completa del genoma del primer caso importado de COVID-19 en un paciente mexicano que había viajado a Bérgamo, Italia	México
Franco, *et al*., 2020 ^31^	Descripción de los patrones de transmisión temprana del virus mediante modelado epidemiológico y la vigilancia del genoma para evaluar las estrategias de mitigación en Panamá	Panamá
Padilla-Rojas, *et al*., 2020a ^32^	Reporte de una secuencia de genoma completo de un paciente peruano con COVID-19 con historial de viaje a Italia	Perú
Padilla-Rojas, *et a*l., 2020b ^33^	Caracterización del genoma completo de SARS-CoV-2 de los primeros casos de COVID-19 en Perú. Se reportan 34 genomas, la mayoría de ellos pertenecientes al clado G linaje B.1.1.1.	Perú
Juscamayta-López, *et al*., 2020 ^34^	Análisis de genomas de SARS-CoV-2 para describir el establecimiento y propagación viral, eventos de transmisión temprana y diversidad genómica	Perú
Poterico, *et al*., 2020 ^35^	Descripción y comparación de posiciones variables a nivel genómico en 30 genomas virales completos de países sudamericanos y de Perú	Perú
Salazar, *et al*., 2020 ^36^	Secuenciación de 10 genomas con genoma completo de 10 SARS-CoV-2 de pacientes diagnosticados durante la introducción viral	Uruguay

## Implementación y aplicaciones de la secuenciación del SARS-CoV-2 en Colombia

Las primeras diez secuencias del SARS-CoV-2 en Colombia se obtuvieron de muestras captadas en ocho ciudades del país (Cartagena, Santa Marta, Ibagué, Bogotá, Medellín, Cali, Palmira y Popayán), donde se confirmaron los primeros casos de COVID-19 importados desde Europa, como parte de la vigilancia virológica de rutina liderada por el Instituto Nacional de Salud de Colombia.

La disponibilidad de protocolos en la web específicos para la secuenciación del SARS-CoV-2 basada en amplicones de PCR ^8^ permitió que los investigadores del Instituto Nacional de Salud, en cooperación con investigadores del Instituto Alexander von Humboldt y la Universidad Cooperativa de Colombia, los aplicaran en la secuenciación del nuevo coronavirus e implementaran la tecnología de secuenciación de Oxford Nanopore - MinION en tan solo tres semanas a partir del primer caso confirmado de COVID-19 en el país, con lo cual las primeras secuencias del SARS-CoV-2 quedaron disponibles en el repositorio de secuencias genómicas del nuevo coronavirus, GISAID. Simultáneamente, investigadores de la Universidad del Valle y del Centro Internacional de Agricultura Tropical demostraron la capacidad instalada para secuenciar el SARS-CoV-2 a partir de muestras captadas localmente ^26^.

Así, las primeras secuencias del SARS-CoV-2 en Colombia evidenciaron la variabilidad genética en diferentes posiciones nucleotídicas de los sitios genómicos del virus, usados en las pruebas moleculares (rRT-PCR) para el diagnóstico de la COVID-19 y disponibles en el sitio web de la Organización Mundial de la Salud (OMS). Ello demostró que la acumulación de mutaciones en el genoma viral es relativamente frecuente y, en consecuencia, es importante evaluar periódicamente los sitios blanco para el diagnóstico molecular usando la información de las secuencias disponibles para verificar mediante simulación por computador su potencial efecto en el desempeño de las pruebas ^9^.

En los meses siguientes otros estudios corroboraron esta información a la luz de nuevas secuencias del SARS-CoV-2 reportadas en GISAID por investigadores colombianos, las cuales se sumaron a las reportadas por otros países de la región, incluidos Brasil, Chile, Ecuador, Uruguay, Perú y Argentina, lo que demuestra el alcance de estos hallazgos en el territorio suramericano ^18^, así como en las pruebas de inmunodiagnóstico basadas en anticuerpos contra las proteínas N y S del SARS-CoV-2 ^10^.

Además, la capacidad de secuenciación instalada del Instituto Nacional de Salud permitió clasificar el primer aislamiento del SARS-CoV-2 colombiano (obtenido por investigadores de la Universidad de Antioquia) en el linaje B.1.5, uno de los más prevalentes en el país. Esta información resulta crucial para determinar las cepas del virus que se emplearán en pruebas *in vitro* para la evaluación de compuestos terapéuticos o desinfectantes ^37^. Por último, las secuencias del SARS-CoV-2 identificadas durante la fase temprana de la pandemia de COVID-19 en Colombia aportaron datos moleculares para respaldar la información epidemiológica relativa a la proporción de casos importados de Europa y América y la reconstrucción de cadenas de transmisión viral, así como para estimar la variabilidad genética de SARS-CoV-2 en el país, traducida en la identificación de 12 sublinajes del SARS-CoV-2 importados en un transcurso de dos meses desde la confirmación del primer caso ^17^.

## Conclusiones y perspectivas

Es una necesidad urgente fortalecer la cooperación entre los laboratorios de salud pública de Latinoamérica y el Caribe para facilitar la transferencia tecnológica, el entrenamiento del personal y la adquisición de recursos para insumos, infraestructura y mejoramiento de la capacidad instalada en el laboratorio, entre otros. Esto permitirá establecer consensos sobre la vigilancia genómica en la región para los sistemas de alerta temprana frente al SARS-CoV-2 y otros agentes patógenos que amenacen la salud pública. Ello debe acompañarse de políticas regionales de asignación de recursos financieros para el mejoramiento de la capacidad instalada en los laboratorios, la adquisición de insumos y equipos y los programas de entrenamiento de profesionales en análisis de datos, lo que permitirá fortalecer los sistemas de vigilancia genómica y de alerta temprana frente a amenazas para la salud pública.
